# Patterns and Drivers of Soil Respiration under Long-Term *Citrus reticulate* in Southern China

**DOI:** 10.1371/journal.pone.0137574

**Published:** 2015-09-14

**Authors:** Yan-Jie Zhang, Su-Yan Zhang, Jie Yang, Yue Yan, Xiang-ping Fu, Shun-Bao Lu

**Affiliations:** 1 Key Laboratory of Poyang Lake Wetland and Watershed Research, Ministry of Education, College of life sciences, Key Lab of Protection and Utilization of Subtropic Plant Resources, Jiangxi Normal University, Nanchang 330022, P. R. China; 2 Jiangxi Research Institute for Soil and Water Conservation, Nanchang 330029, P. R. China; 3 College of Foreign Languages, Jiangxi Agricultural University, Nanchang 330022, P. R. China; Tennessee State University, UNITED STATES

## Abstract

Soil respiration (*Rs*) is a major source of carbon emission in terrestrial ecosystems. Despite the fact that the influence of land use practice on *Rs* has been widely studied, the patterns and drivers on *Rs* of *Citrus reticulata* cultivation, a worldwide land use practice are unclear. In this current study, we investigated the influence of long-term cultivation of *Citrus reticulata* (CO) and of CO intercropped with soybean (CB) on soil nutrients, water availability, and *Rs* in southern China. Results indicated that after 21 years of cultivation, CO and CB significantly increased total soil carbon (TC), total soil nitrogen (TN), and soil organic matter (OM) at 0–20 cm and 20–40 cm, both at upslope and downslope compared with bare soil (CK). However, soil moisture (SM), dissolved organic carbon (DOC), and microbial biomass carbon (MBC) decreased under CB. In addition, no significant variation was found in soil pH between CK, CO, and CB. Across incubation time (56 days), *Rs* decreased exponentially with incubation time and CB showed the highest *Rs* rate irrespective of soil depth or topography. Linear regression further showed TC and TN as the two major factors influencing *Rs* upslope, while DOC was the dominant factor in regulating *Rs* downslope. These findings demonstrated that long-term cultivation of citrus significantly changed soil nutrients, water availability, and *Rs* rate.

## Introduction

Soil respiration (*Rs*) is a major source of carbon emission in terrestrial ecosystems and plays an important role in the global carbon cycle [1]. It is estimated that the global flux of CO_2_ from *Rs* is approximately 68×10^15^ g C/year [2], which is higher than the carbon fixed by terrestrials ecosystems (i.e., 56.4×10^15^ g C/year). The higher carbon emission from *Rs* indicates that even a small change in *Rs* rate could trigger a profound effect on global carbon cycling [1,3,4]. It is widely reported that land use practice could dramatically change *Rs* rates, however, the patterns and drivers of *Rs* on *Citrus reticulata* cultivation are still not well understood.

Worldwide, cultivation of *C*. *reticulata* is a common land use practice and its cultivated area increased dramatically in recent years due to its economic value [5]. For example, in China alone, the cultivated area of *C*. *reticulata* has been increased 10 times during the past 30 years, with a total cultivated area of 19.1×10^5^ hm^2^ in 2006 [6]. It has been reported that cultivation of *C*. *reticulata* could greatly alter *Rs* rate, but the changed magnitude varied considerably among different studies (i.e., ranging from 6.98 to 10.1 ×10^6^ g C/hm^2^ year), even among cultivated *C*. *reticulata* under similar climatic conditions and similar cultivation history [7,8]. Previous studies exclusively attributed the varied *Rs* rate to changed soil temperature induced by cultivation of *C*. *reticulata*, while the potential contribution of soil nutrients and water availability was neglected[7,8]. Recent studies have shown that soil nutrients and water availability also changed significantly under long-term cultivation of *C*. *reticulata*, indicating that besides soil temperature, soil nutrients and water availability also play an important role in determining *Rs* rate variation in *C*. *reticulata* plantations.

In particular, long-term cultivation of *C*. *reticulata* directly increased soil organic carbon (SOC) and total nitrogen (TN) via the input of soil organic matter (OM) from litter decomposition and below-ground biomass turnover [9,10]. For example, it has been reported that mature *C*. *reticulata* could produce 16.82 × 10^5^ g litter /hm^2^ year and nearly 80% of this litter was decomposed within one year [11]. Moreover, field observations also reported that mature *C*. *reticulata* could reduce runoff by about 33%-95% due to the high water-holding rate of soil caused by citrus litter [12]. Despite numerous studies have demonstrated that *Rs* was also sensitive to soil nutrients and water availability [7,13], the contribution of soil nutrients and water availability to *Rs* variation in *C*. *reticulata* cultivation has not been fully investigated.

The long-term cultivated *C*. *reticulata* orchards in southern China provided an ideal target to examine the influence of patterns and drivers (soil nutrients and water availability) on *Rs* under *C*. *reticulata* because the cultivation history and density in this area was clearly recorded. In addition, southern China has the largest cultivated citrus area in the world and *C*. *reticulata* in this area is often intercropped with other crops (i.e., soybean), which provided an opportunity to comprehensively examine the influence of *C*. *reticulata* on *Rs*[12]. In this study, we tried to address the following two questions: first, how do soil nutrients, water availability and *Rs* change under long-term *C*. *reticulata* cultivation? Second, how do soil nutrients and water availability regulate *Rs* under long-term *C*. *reticulata* cultivation?

## Materials and Methods

### 2.1 Study site

This study was conducted in the Ecological and Technological Station of Water and Soil Conservation (ETSWC) in Jiujiang city, Jiangxi province, southern China (115°42′38″E, 29°16′37″N). This study did not involve any endangered or protected species. ETSWC was established in 1991, covering a total area of approximately 80 ha, with an average slope of 12°. Altitude in the area decreased from 90 m in northwest to 30 m in sourtheast. The soil was classfied as hapludult based on the USDA Soil Taxonomy [14,15]. ETSWC was characterized by a subtropical monsoon climate [12]. Local meteorological data (2001–2010) showed a mean annual precipitation of 1469 mm and a mean annual temperature of 16.7°C, with the lowest mean monthly temperature in January (3.5°C) and the highest in July (29.6°C) [12].

### 2.2 Experimental design

To test the influences of different land use practices on ecosystem function, we carried out a field experiment in ETSWC beginning in 1991. In particular, we selected a slope with relatively uniform vegetation and cleared all plants. In the initial design, there were six different land use practices. In this study, we selected three typical land use practices to examine their influence on *Rs* under long-term *C*. *reticulata* cultivation: bare soil maintained by artificial weeding in every year (CK), cultivating citrus alone (*C*. *reticulata*) with the citrus density of 1,200 individual per ha (CO), and citrus intercropped with *Glycine max* with the soybean density of 62,500 individual per ha (CB). These land use practices were randomly arranged in nine 5 m×20 m block with three replicates for each land use practice. These blocks were established in 1991 and these three land use practices continued for 21 years until this study was conducted in 2011.

### 2.3 Soil sampling

We collected soil samples from each replicate in each land use practice in October 2011. A previous study showed that elevation had a great impact on soil nutrient and water availability under *C*. *reticulata* cultivation [10]. Thus, in each land use practice, we took soil samples from two different elevations: one soil sample was taken from upslope and another from downslope, with an approximately 4-meter elevation difference between upslope and downslope positions. At each elevation, we took soil cores from 5 randomly-determined locations at a depth of 0–20 cm and 20–40 cm using a 7.5 cm diamater soil auger. Then, soil cores collected from the same depth were mixed *in situ* into one composite sample. A total of 18 soil samples was collected from all blocks. The collected soil samples were immediately placed into a portable cooler and transported to a laboratory for further analysis.

### 2.4 Soil respiration and properties measurement

After removing litters and roots and sieving through 2-mm mesh, the collected soil samples were then divided into two subsamples. One subsample was used to measure *Rs* through the alkali absorption method. First, 30 g fresh soil was adjusted to 60% of the field water holding capacity. Second, the adjusted soils were aerobically incubated at 22°C in a 1L sealed glass jar. CO_2_ that evolved from soils was trapped in 0.1 M NaOH and measured by 0.1 M HCl titration after 1, 3, 7, 14, 21, 28, 35, 42, 49, and 56 days [16]. The total *Rs* was estimated by calculating the cumulative production of CO_2_ from soils during the 56 incubated days.

Meanwhile, the other subsample was used to measure soil nutrient and water availability: total carbon (TC), TN, OM content, dissolved organic carbon (DOC), microbial biomass carbon (MBC), soil moisture (SM), and pH. TC and TN were measured by elemental analyzer (Isoprime- EuroEA3000, Milan Italy). OM, DOC, MBC, SM, and pH were measured by potassium dichromate oxidation- outer heating method, high-temperature catalytic oxidation method, chloroform fumigation extraction method, oven drying method, and potentiometric method, respectively.

### 2.5 Data analysis

Statistical analysis was carried out using SPSS 13.0 for Windows (SPSS Inc., Chicago, IL). Two-way ANOVA followed by Fisher’s least significant differnce (LSD) test was used to examine the effects of land use practice, soil depth, and their interactive effects on soil properties. Repeated measures ANOVA (RMANOVA) was used to examine *Rs* variability across days. Linear regression was used to examine relationship of *Rs* with soil nutrient and water availability.

## Results

### 3.1 Response of soil nutrient and water availability to long-term *C*. *reticulata* cultivation

**Fig 1:**
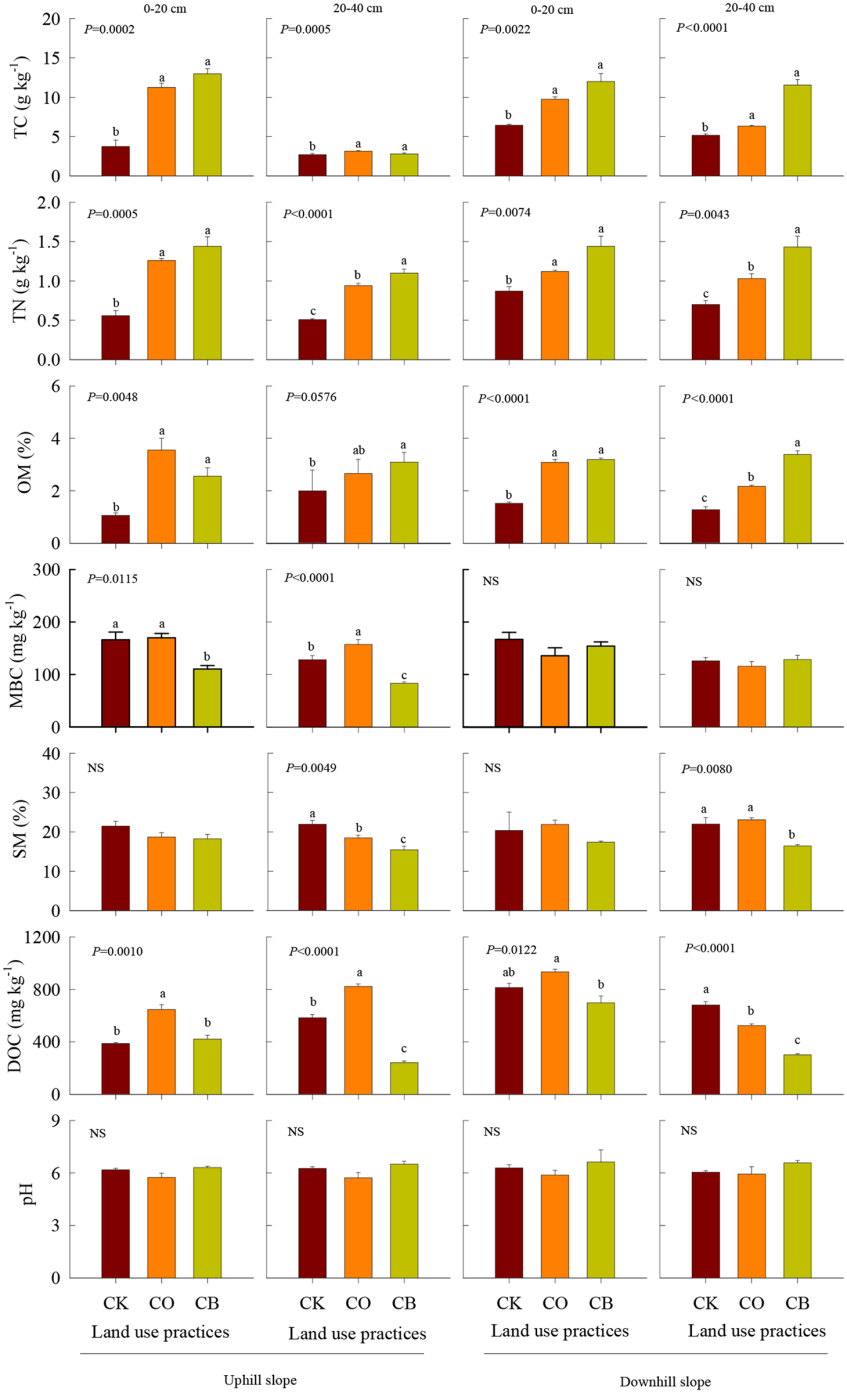
Response of total carbon (TC), total nitrogen (TN), organic matter (OM), soil moisture (SM), dissolved organic carbon (DOC), microbial biomass carbon (MBC) and pH (mean±1SD) to bare soil (CK), citrus cultivation only (CO), and citrus intercropped with soybean (CB).

Land use practices, soil depth, and their interaction had significant effects on TC, OM, and DOC content both in upslope and in downslope (Table 1). Under long-term *C*. *reticulata* intercropped with *G*. *max* (CB), TC increased by 346% and 116% at 0–20 cm and 20–40 cm in upslope and 186% and 166% in downslope compared to CK, respectively (Fig 1). OM increased by 241% and 155% at 0–20 and 20–40 cm in upslope and 121% and 263% in downslope compared to CK, respectively (Fig 1). Compared with CK, TC and OM at 0–20 cm for equivalent slope positions was higher than that at 20–40 cm in all three treatments (Fig 1). However, DOC decreased by 108% and 41.5% under CO and under CB at 20–40 both at upslope and downslope relative to CK, respectively (Fig 1).

**Table 1:** P values of analysis of variance for soil nutrients and water availability using land use practices (L), soil depth (D), and their interaction as fixed effects.

Soil properties	Terms	*df*	Uphill slope	Downhill slope
TC	L	2	<0.0001****	<0.0001****
	D	1	<0.0001****	0.0019**
	L*D	2	0.0085**	0.0419*
TN	L	2	<0.0001****	<0.0001****
	D	1	0.0005***	0.231
	L*D	2	0.0663	0.666
OM	L	2	0.0002***	<0.0001****
	D	1	0.0271*	0.0013**
	L*D	2	0.0034**	0.0003***
SM	L	2	0.0013**	0.0505
	D	1	0.2981	0.7229
	L*D	2	0.2678	0.8057
DOC	L	2	<0.0001****	<0.0001****
	D	1	0.0049**	<0.0001****
	L*D	2	<0.0001****	0.0007***
MBC	L	2	<0.0001****	0.1605
	D	1	0.0041**	0.0053**
	L*D	2	0.3956	0.5995
pH	L	2	0.79	0.201
	D	1	0.5648	0.774
	L*D	2	0.8571	0.916

TC, TN, OM, SM, DOC, and MBC represented total carbon, total nitrogen, organic matter, soil moisture, dissolved organic carbon, and microbial biomass carbon content, respectively. Significant differences between soils from different treatments are indicated as:

*, *P* < 0.05;

**, *P* < 0.01;

***, *P* < 0.001;

****, *P* < 0.0001.

Meanwhile, land use practice and soil depth had significant main effects on TN and SM (Table 1). Compared with CK, TN under CB increased by 257% and 216% at 0–20 cm and 20–40 cm in upslope and increased by 166% and 205% in downslope. Relative to CK, SM significantly decreased under CB only at 20–40 cm both at upslope and at downslope while MBC significantly decreased under CB only at upslope compared to CK (Fig 1). For equivalent slope position, no significant variation was detected in soil pH at each soil depth and at each topographic position (Table 1, Fig 1).

### 3.2 Response of *Rs* to long-term *C*. *reticulata* cultivation

**Fig 2:**
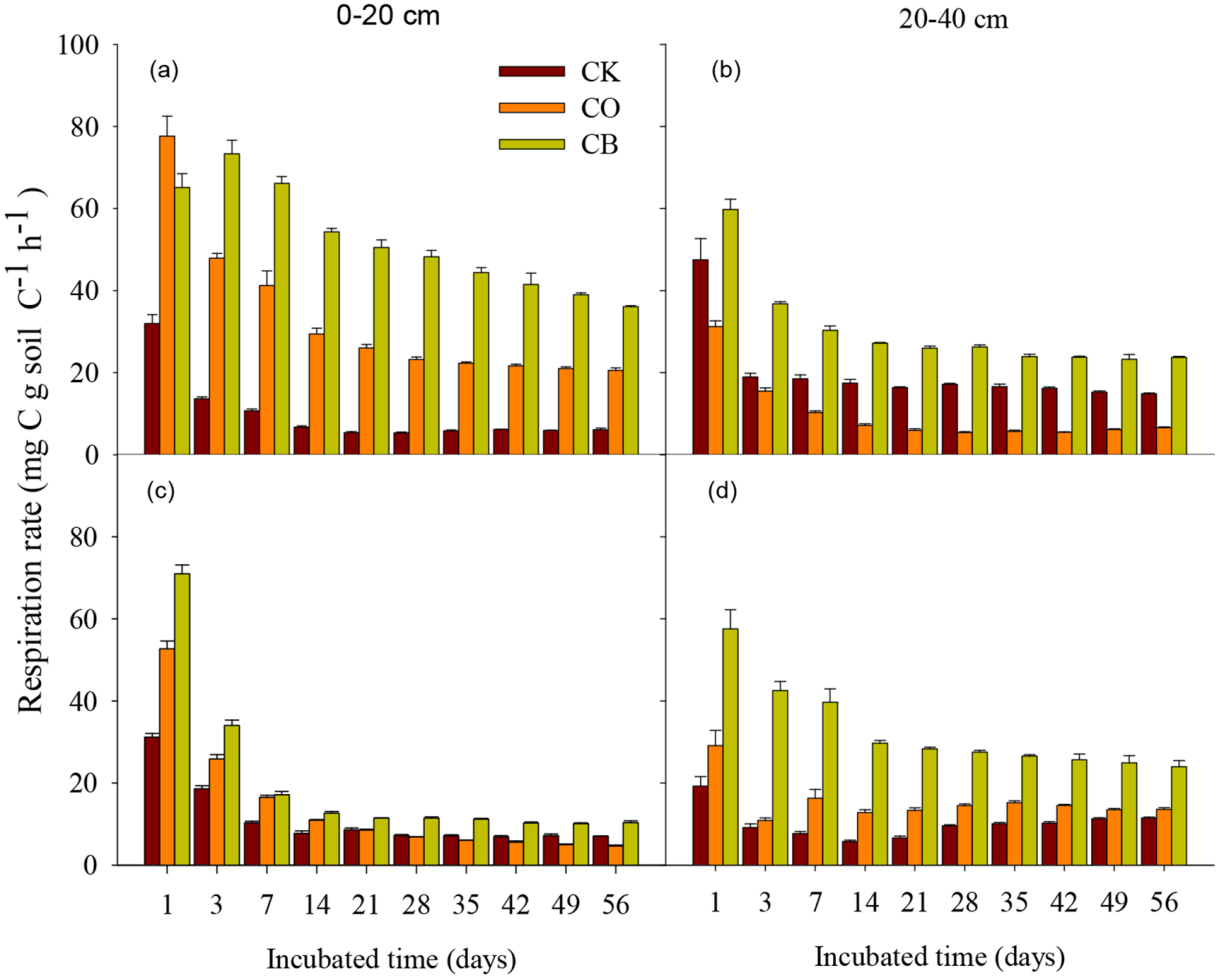
Variation of soil respiration (mean±1SD) at uphill (a,b) and downhill slope (c,d) across incubation time.

Results from RMANOVA showed that land use practices and land use practices × time had a significant effects on *Rs* both at 0–20 cm and 20–40 cm for equivalent slope position (Table 2). For all land use practices, *Rs* decreased exponentially with incubation time except CK and CO at 20–40 cm at downslope (Fig 2), with the highest *Rs* at 1 day (ranged from 19.23 to 77.65 mg C/g soil C h) and lowest *Rs* at 56 day (ranging from 6.07 to 36.08 mg C/g soil C h) under all three treatments. At each incubation time, CB had the highest *Rs* rate irrespective of soil depth and topography (Fig 2). At 0–20 cm, the average *Rs* for CB at upslope was 51.86 mg C/g soil C h, which was 1.56 and 5.30 times higher than that for CO and CK at upslope, respectively. The average *Rs* for CB at downslope was 30.10 mg C/g soil C h, which was 3.03 and 1.51 times higher than that for CO and CK at downslope, respectively.

**Table 2:** P-values of repeated measures analysis for soil respiration using land use practices (L) as between-subject effect and incubation time (T) as within-subject effects.

Terms	*df*	Uphill slope		Downhill slope	
		0–20 cm	20–40 cm	0–20 cm	20–40 cm
L	2	<0.0001	<0.0001	<0.0001	<0.0001
T	9	<0.0001	<0.0001	<0.0001	<0.0001
L*T	18	<0.0001	<0.0001	<0.0001	<0.0001

**Fig 3:**
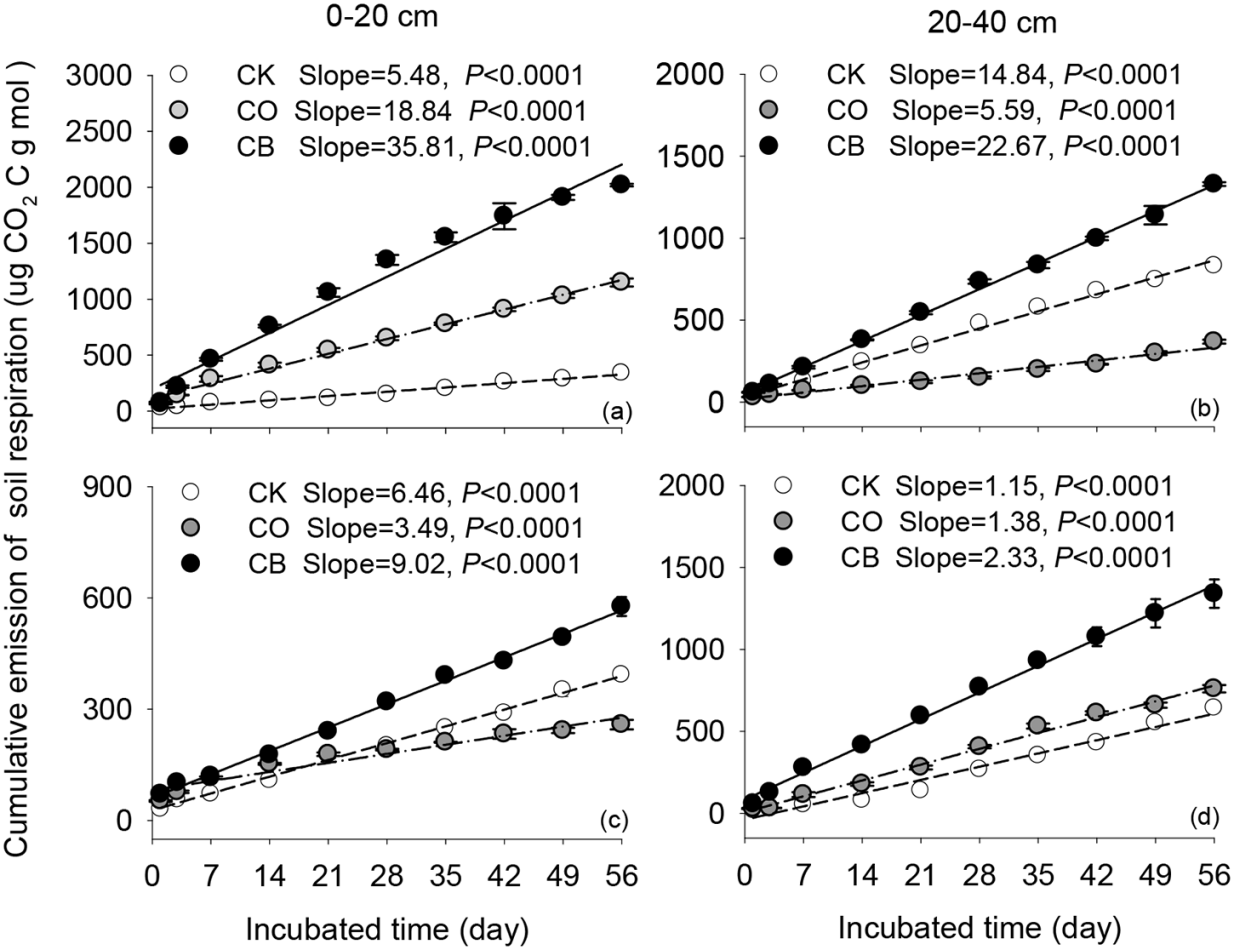
Relationship between cumulative carbon emission fromsoil respiration (mean±1SD) with incubation time (day) at uphill (a,b) and downhill slope (c,d). Dashed, dashed with dot, and solid lines represented the fitted relationship of soil respiration under bare soil (CK), cultivation of citrus (CO), and citrus intercropped with soybean (CB) with incubation time, respectively.

During the whole incubation time, the cumulative carbon emission from *Rs* was the highest under CB, ranging from 577.0 to 2020.4 μg CO_2_-C/g dry soil under CB (Fig 3). The cumulative emission was the lowest under CK at 0–20 cm at upslope and at 20–40 cm at downslope (Fig 3). Results from linear regression showed that the cumulative carbon emission from *Rs* was positively correlated with incubation time but a significant difference was detected between slopes (all *p*<0.05) (Fig 3). Among these three land use practices, CB had the highest cumulative slope irrespective of soil depth and topography (all *p*<0.05) (Fig 3).

### 3.3 Relationship between *Rs* and soil nutrients and moisture under long-term *C*. *reticulata* cultivation

**Fig 4:**
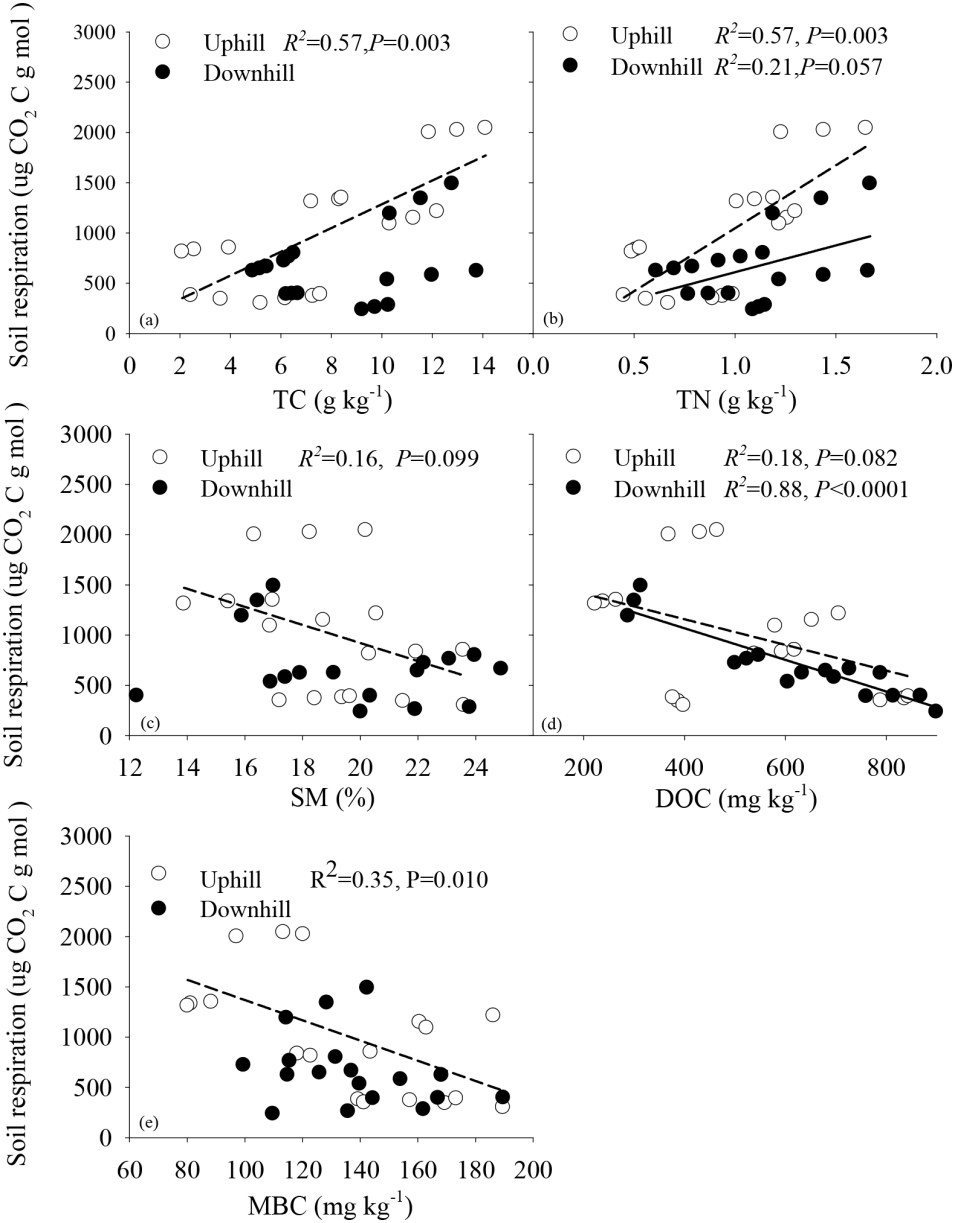
Relationship between the cumulative carbon emission from soil respiration and soil nutrients and water availability. TC, TN, SM, DOC, and MBC represented total carbon, total nitrogen, soil moisture, and microbial biomass carbon content, respectively. Dashed and solid lines represented the fitted lines between soil respiration and soil properties at uphill and downhill slope, respectively.

At equivalent slope position, we pooled soil nutrient and water availability at 0–20 cm and 20–40 cm together and then analyzed their relationship with cumulative carbon emission from *Rs* due to small sample sizes for individual soil depths. At upslope, the cumulative carbon emission from *Rs* was posivelty correlated with total carbon (TC) and total nitrogen (TN) (Fig 4a and 4b) but negatively correlated with SM, DOC and MBC (Fig 4c and 4e). TC, TN, SM, DOC, and MBC alone explained 57%, 57%, 16%, 18%, and 35% of total variation in the cumulative carbon emission from *Rs*, respectively (Fig 4a and 4e). At downslope, the cummulative carbon emission from *Rs* was also posivelty correlated with TN and negatively correlated with DOC (Fig 4b and 4d). TN and DOC alone accounted for 21% and 88% of total variation in the cummulative carbon emission from *Rs* (Fig 4b and 4d). No significant interactive effects on *Rs* were found between TC, SM, MBC, and the cumulative carbon emission from *Rs* at downslope (Fig 4a, 4c and 4e).

## Discussion

### 4.1 Response of soil nutrients and water availability to long-term *C*. *reticulata* cultivation

Our results showed that after 21 years of treatment, long-term citrus cultivation significantly increased TC, TN, and OM under CO and CB compare to CK, which was consistent with previous studies [10]. This could be explained by the following possibilities. First, litter decomposition was an important source of nutrients that increased soil TC, TN, OM, and DOC. It was reported that mature *C*. *reticulata* could produce 16.82 × 10^5^ g litter /hm^2^ year and nearly 80% of the litter was decomposed within 1 year [11]. Second, the high temperature and moist climate in this area cause citrus to have a high fine root turnover rate (i.e., 0.852/year) [17], which in turn can increase soil TC, TN, and OM [16]. Finally, the intercropped soybean can also fix atmospheric N and result in an increase of soil nutrient availability [18].

Our results also demonstrated that soil pH showed no significant response to different plantation treatments, indicating that long-term citrus cultivation do not lead to significant soil acidification in our experiment. This findings was inconsistent with previous studies as it is revealed long-term citrus cultivation would lead significant soil acidification [19]. In one previous study, the accumulated H^+^ ions from nitrogen fertilizer were believed to be the dominant factor that contributed to the decreased soil pH under *C*. *reticulata* cultivation [19]. In our study, increased soil organic matter may have buffered the effect of soil acidification caused by increased H^+^ ions, which in turn resulted in no significant variation detected in soil pH [20].

### 4.2 Response of *Rs* to long-term *C*. *reticulata* cultivation

It was reported that in a subtropical area, *Rs* under *C*. *reticulata* cultivation was higher than that under other fruit tree species (i.e., *Castanea* and *Pinus*) [8, 21, 22] but lower than that in the climax vegetation (i.e., subtropical evergreen broadleaved forest) [7], indicating that long-term citrus plantation had a great impact on soil carbon emission. In our study, *Rs* under citrus intercropped with soybean was significantly higher than that in citrus cultivation alone, which indicated that citrus intercropped with soybean accelerated soil carbon emission.

### 4.3 Relationship between *Rs* and soil nutrients and moisture under long-term *C*. *reticulata* cultivation

Previous studies have shown the effects of soil nutrients and water availability on *Rs* were still unclear [7,8]. In this study, we found that TC, TN, SM, DOC, and MBC at upslope also played important roles in regulating *Rs* under long-term *C*. *reticulata* cultivation. The positive correlation between TC,TN, and *Rs* in this study was in agreement with previous findings because microbial activity and microbial respiration had been shown to be depended on the supply of TC and TN [23,24]. Meanwhile, we found negative correlation between SM and *Rs* because in south China, abundant rainfall ensured that soil moisture is not the limited factor in influence *Rs*. Contrarily, higher soil moisture will decrease oxygen content in soil, which in turn produce a decrease in *Rs* [25]. The negative correlation between DOC, MBC, and *Rs* in our study was contradictory to previous results in this area [26]. We are unclear on the reason for this negative correlation, and further studies are warranted to find the potential reasons.

Among these soil factors, we found that TC and TN were two major factors that influenced the *Rs* at upslope as they explained more than half of total variation in *Rs*. However, at downslope, DOC was the dominant factor that regulated *Rs* because DOC alone explained 88% of total variation. These findings indicated that, under long-term *C*. *reticulata* cultivation, the relationship between *Rs* and soil nutrients and water availability was also regulated by topography.

## Conclusions

Long-term cultivation of *C*. *reticulata* had significant effects on soil nutrients and water availability and *Rs*. Compared with CK, CO and CB significantly increased TC, TN, and OM at 0–20 cm and 20–40 cm both at upslope and downslope but had no significant effect on soil pH. Meanwhile, SM and MBC decreased under CB. For all these land use practices, CB had the highest *Rs* rate irrespective of soil depth and topography. TC and TN were two major factors that influenced *Rs* upslope while DOC was the dominant factor that regulated *Rs* downslope.
